# Financial burden of pediatric firearm-related injury admissions in the United States

**DOI:** 10.1371/journal.pone.0252821

**Published:** 2021-06-23

**Authors:** Jordan S. Taylor, Sriraman Madhavan, Ryan W. Han, Julia M. Chandler, Lakshika Tenakoon, Stephanie Chao

**Affiliations:** 1 Department of Surgery, Division of Pediatric Surgery, Stanford University, Stanford, California, United States of America; 2 Department of Statistics, Stanford University, Stanford, California, United States of America; 3 Department of Computer Science, Stanford University, Stanford, California, United States of America; 4 Department of Surgery, Division of General Surgery, Stanford University, Stanford, California, United States of America; Kaohsuing Medical University Hospital, TAIWAN

## Abstract

Pediatric firearm-related injuries pose a significant public health problem in the United States, yet the associated financial burden has not been well described. This is the first study examining national data on the cost of initial hospitalization for pediatric firearm-related injuries. In this retrospective review, the Healthcare Cost and Utilization Project Kids’ Inpatient Database from the years 2003, 2006, 2009, and 2012 was used to identify all patients 18 years of age and under who were admitted with firearm-related injuries. We compared demographic and discharge-level data including injury severity score, hospital length of stay, income quartile, injury intent, and inflation-adjusted hospital costs across age groups (0–5, 6–9, 10–15, 16–18 years). There were approximately 4,753 pediatric firearm-related admissions each year, with a median hospitalization cost of $12,984 per patient. Annual initial hospitalization costs for pediatric firearm injuries were approximately $109 million during the study period. Pediatric firearm-related injuries predominately occured among older teenagers (74%, 16–18 years), males (89%), black individuals (55%), and those from the lowest income quartile (53%). We found significant cost variation based on patient race, income quartile, injury severity score, intent, hospital length of stay, disposition, and hospital region. Inflation-adjusted hospitalization costs have increased significantly over the study period (p < 0.001). Pediatric firearm-related injuries are a large financial burden to the United States healthcare system. There are significant variations in cost based on predictable factors like hospital length of stay and injury severity score; however, there are also substantial discrepancies based on hospital region, patient race, and income quartile that require further investigation.

## Introduction

It has been over two years since the deadliest mass shooting in United States history when 58 people were killed and 851 injured at a Las Vegas concert. In 2020, there were over 10,000 non-suicide deaths and 20,000 injuries attributed to guns, including more than 2,800 children < 17 years old who were killed or injured [1]. Compared to other high-income countries, the U.S. firearm-related fatality rate is 49 times higher for young adults ages 15–24 [2]. Globally, nine out of every 10 children <15 years of age killed by firearms resided within the U.S [2]. In 2016, firearm-related injuries, inclusive of homicide, suicide, and unintentional injury, were second only to motor vehicle collisions as the leading cause of death in the U.S. pediatric population [3].

The cost of these injuries has not been well studied. In the U.S., the overall cost of firearm injuries is estimated to be $734 million per year, with the burden of payment falling primarily on governmental insurance programs [4]. This number fails to capture the actual economic impact of firearm-related injuries, with societal costs estimated to be greater than $174 billion annually when work-loss, mental health, and quality of life costs are considered [5]. In children and young adults < 21 years of age, firearm injuries were estimated to have a financial cost of upwards of $330 million in 2010, with the majority due to hospitalization costs for nonfatal injuries [6]. In the same year, the combined medical and work-loss cost for all fatal firearm injuries among children was estimated to be over $4.8 billion [3]. Nearly 50% of children hospitalized with firearm-related injuries are discharged with a disability, limiting their ability to pursue education and participate in the workforce, suggesting an enormous economic impact as a result of these injuries [7].

Our objective was to characterize the cost of initial hospitalization from pediatric firearm-related injury and mortality over time and to identify factors that may contribute to variation in cost. Improved understanding of the costs of firearm injuries can help inform public policy.

## Methods

### Data source

Data on firearm-related injuries and costs were obtained from the Kids’ Inpatient Database (KID), provided by the Healthcare Cost and Utilization Project (HCUP), which is sponsored by the Agency for Healthcare Research and Quality [8]. The KID is the largest publicly available all-payer pediatric inpatient care database in the U.S., sampling up to 80% of pediatric discharges from more than 4,100 U.S. hospitals. Released every three years, KID yields national measures of hospital inpatient stays for patients younger than 21 years of age, as well as national population data by age and year to allow for estimates of incidence. It contains data from approximately 3 million pediatric discharges annually but can be weighted to achieve national estimates of approximately 7 million discharges. For the present study, data from 4 reporting years were used: 2003, 2006, 2009, and 2012. During the years examined, there were between 36–44 states represented depending on the year.

### Study population

Patients who met inclusion criteria were identified using the International Classification of Diseases, 9th Revision (ICD-9) codes for admissions due to firearm-related injuries. Patients with the following ICD-9 diagnosis codes were included: E922.0-.3,.8-.9; E955.0-.4; E965.0-.4; E979.4; E985.0-.4; or E970. Because E-codes identify emergency conditions, we were able to identify injured patients during their initial hospitalization. Of note, the KID only captures inpatient hospitalization, thus we were not able to include patients treated and released from an emergency department. KID also does not link subsequent hospitalizations by patient identifiers, therefore readmission data, outpatient visits, and rehabilitation facility care were not captured by the present study.

All patients 18 years of age and under were included in our analysis, and admissions were grouped based on four age ranges (0–5, 6–10, 11–15, 16–18 years). Race and ethnicity were reported according to the KID classifications, which include White, Black, Hispanic, Asian/Pacific Islander, Native American, and Other.

### Outcome measures

The primary outcome of interest was the cost associated with initial admissions for pediatric firearm-related injuries. In order to compare costs, we inflation-adjusted costs to 2018 values using the Consumer Price Index rates. The secondary aim was to compare characteristics of those injured by firearms (i.e. age, race, injury severity, intent of injury) based on age group.

### Statistical analysis

To calculate incidence rates and costs that were reflective of estimates for the entire U.S. pediatric population, we applied survey weights according to HCUP recommendations [9]. We used the Wilcoxon rank-sum test and Kruskal-Wallis test to compare costs between groups with two and three or more levels respectively. Chi-squared analysis was used to compare the incidence distribution between groups. The Mann-Kendall test was used to detect a trend in costs. P values less than 0.05 were considered significant, and Bonferroni corrections were applied to account for multi hypothesis testing. We used R version 4.0.0 (R Foundation for Statistical Computing, Vienna, Austria) for analysis.

## Results

A total of 19,015 pediatric patients 18 years of age and under were admitted for firearm-related injuries during the study period, with an average of 4,753 admissions per year. The overall incidence has declined from its peak of 5,858 in 2006 to 3,828 in 2012. The oldest age group (16–18) accounted for 74% of all admissions. The majority of patients were male (89%), black (55%), or from the lowest income quartile (53%). Assault was the most common intent, accounting for 63% of admissions; unintentional injuries accounted for 26% of admissions; self-inflicted injuries accounted for only 3% of inpatient hospitalizations. Nearly half (47%) of the patients had a hospital length of stay (LOS) of four or more days.

**Fig 1:**
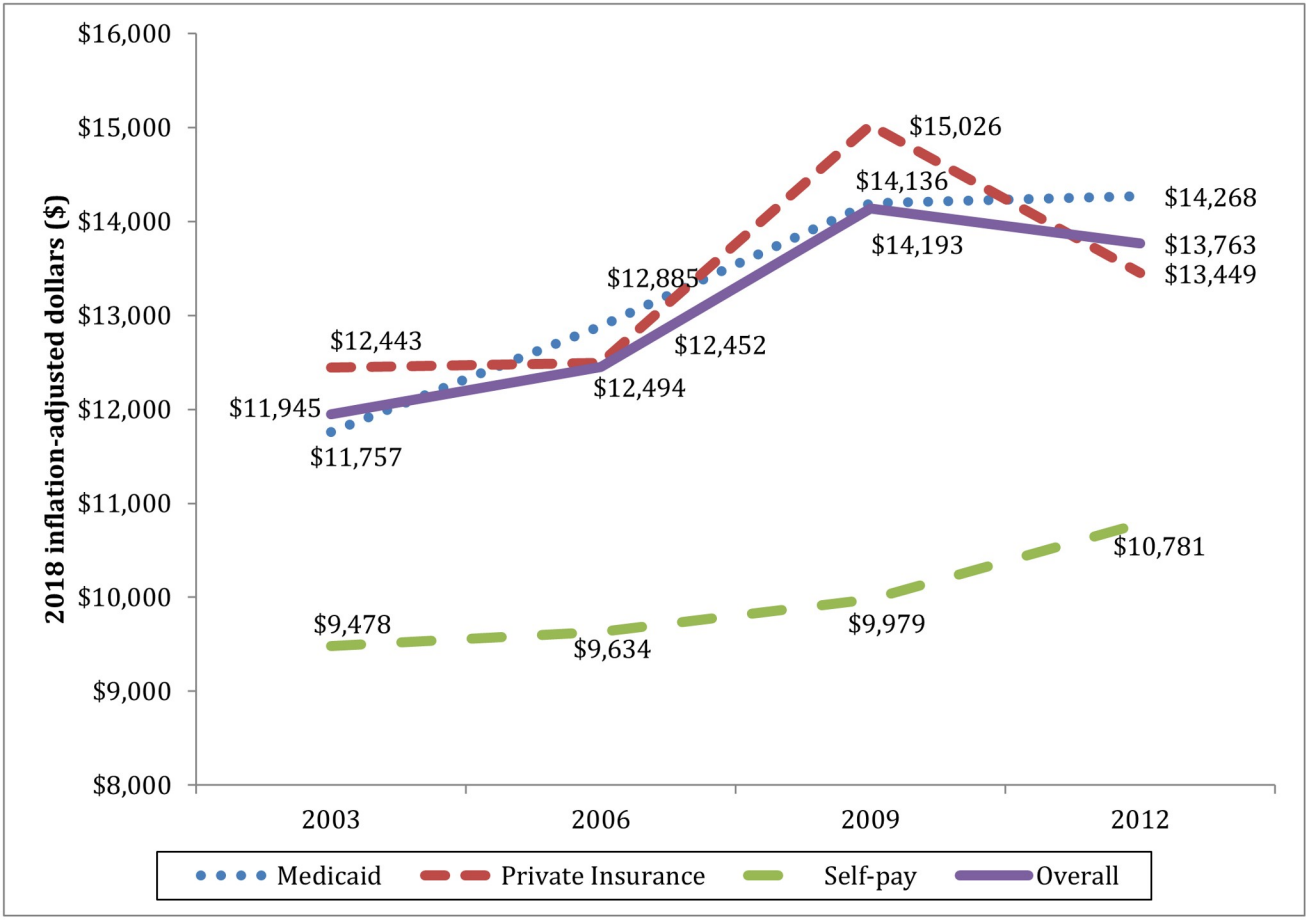
Average hospital cost per admission for pediatric firearm-related injuries by payer type. All data from 2003–2012.

Initial hospitalization for pediatric firearm injuries cost the US health care system $109 million per year, with a median cost of $12,983 and first and third quartile of $6,387 and $25,885 respectively. There was a significant increase in inflation-adjusted costs over the time period (p < 0.001), although the difference between 2009 and 2012 was not significant (Fig 1). While the median cost for Medicaid and self-paying patients increased between 2009 and 2012, the median cost for privately insured patients fell by $1,577 per admission.

On unadjusted analysis, several variables were found to be significantly associated with the cost of hospitalization (Table 1). A higher injury severity score (ISS), increased LOS, and higher income quartile were all associated with higher costs (p < 0.001). Race, intent of injury, and payer type were also significantly associated with variability in hospitalization costs. Asian race, while only accounting for 1.2% of total injuries, had the highest median hospitalization cost at $18,983 per admission. Unintentional injury admissions had a median cost of $10,999 while the median cost of self-inflicted injuries and injuries resulting from legal intervention were $15,323 and $14,985, respectively. Self-pay patients had the lowest median cost at $9,878. There was no significant difference in cost by age group or gender. Cost varied significantly by hospital region (p < 0.001), with the West having the highest median cost of $18,244 (Table 1). However, upon controlling for ISS, LOS, and hospital region, the above cost differences lost their significance, while ISS, LOS, and hospital region remained significant.

**Table 1:** Incidence and median cost of pediatric firearm hospitalizations in the U.S. (2003, 2006, 2009, 2012).

Dimension	Annual cases n (%)	Hospitalization costs	
		Median admission cost (IQR), thousands of $	P
*Demographic factors*			
**Race**			<0.001
White	661 (17.1%)	14.1 [6.8, 29.0]	
Black	2138 (55.1%)	12.6 [6.0, 24.8]	
Hispanic	861 (22.2%)	15.2 [7.5, 33.0]	
Asian	45 (1.2%)	19.0 [9.8, 37.8]	
Native American	27 (0.7%)	11.1 [6.2, 22.6]	
Other	145 (3.7%)	14.2 [7.1, 27.6]	
**Age, years**			0.790
0–5	125 (2.6%)	13.3 [6.4, 31.3]	
6–10	146 (3.1%)	11.5 [5.5, 24.1]	
11–15	976 (20.5%)	12.6 [6.0, 25.8]	
16–18	3507 (73.8%)	13.2 [6.6, 25.7]	
**Gender**			1
Male	4153 (88.5%)	13.0 [6.4, 25.8]	
Female	538 (11.5%)	13.9 [6.4, 27.4]	
**Hospital region**			<0.001
Northeast	575 (15.8%)	9.8 [4.6, 19.2]	
Midwest	925 (25.5%)	12.8 [6.3, 23.3]	
South	1194 (32.9%)	12.8 [6.5, 24.5]	
West	938 (25.8%)	18.2 [9.2, 38.9]	
**Payer**			<0.001
Medicaid	2578 (54.5%)	13.1 [6.4, 26.8]	
Private Insurance	1322 (27.9%)	13.5 [6.8, 25.9]	
Self-Pay	542 (11.5%)	9.9 [5.1, 17.9]	
Other	263 (5.6%)	16.5 [7.4, 32.3]	
**Income quartile**			<0.001
1	2445 (53.1%)	12.2 [6.0, 24.0]	
2	1138 (24.7%)	13.6 [7.0, 26.5]	
3	706 (15.3%)	14.4 [7.2, 28.8]	
4	317 (6.9%)	16.2 [7.6, 33.3]	
*Injury specific factors*			
**ISS**			<0.001
0–9	2944 (61.9%)	9.3 [4.7, 17.1]	
10–15	821 (17.3%)	17.7 [10.3, 29.8]	
16–25	765 (16.1%)	24.7 [13.6, 50.8]	
> 26	224 (4.7%)	37.7 [20.5, 74.2]	
**Intent**			<0.001
Unintentional	1173 (26.2%)	11.0 [5.6, 21.8]	
Self-inflicted	142 (3.2%)	15.3 [7.7, 33.4]	
Assault	2836 (63.3%)	13.4 [6.6, 26.2]	
Undetermined	291 (6.5%)	12.7 [5.8, 23.5]	
Legal Intervention	38 (0.8%)	14.9 [7.2, 47.5]	
**LOS, days**			<0.001
0	398 (8.4%)	5.2 [2.8, 10.4]	
1	934 (19.7%)	5.4 [3.1, 9.4]	
2	688 (14.5%)	8.0 [5.0, 12.3]	
3	491 (10.3%)	10.7 [7.3, 15.7]	
4+	2241 (47.2%)	24.7 [15.2, 44.5]	
**Disposition**			<0.001
Routine	3658 (77.2%)	5.6 [11.2, 21.4]	
Transfer to short-term hospital	142 (3%)	23.8 [10.0, 53.2]	
Transfer to other facility	291 (6.1%)	41.1 [19.4, 82.7]	
Home health care	294 (6.2%)	22.7 [12.4, 42.6]	
Left AMA	38 (0.8%)	8.4 [4.1, 16.9]	
Died in hospital	317 (6.7%)	15.8 [9.3, 29.8]	

ISS, injury severity score; LOS, length of stay; AMA, against medical advice

**Fig 2:**
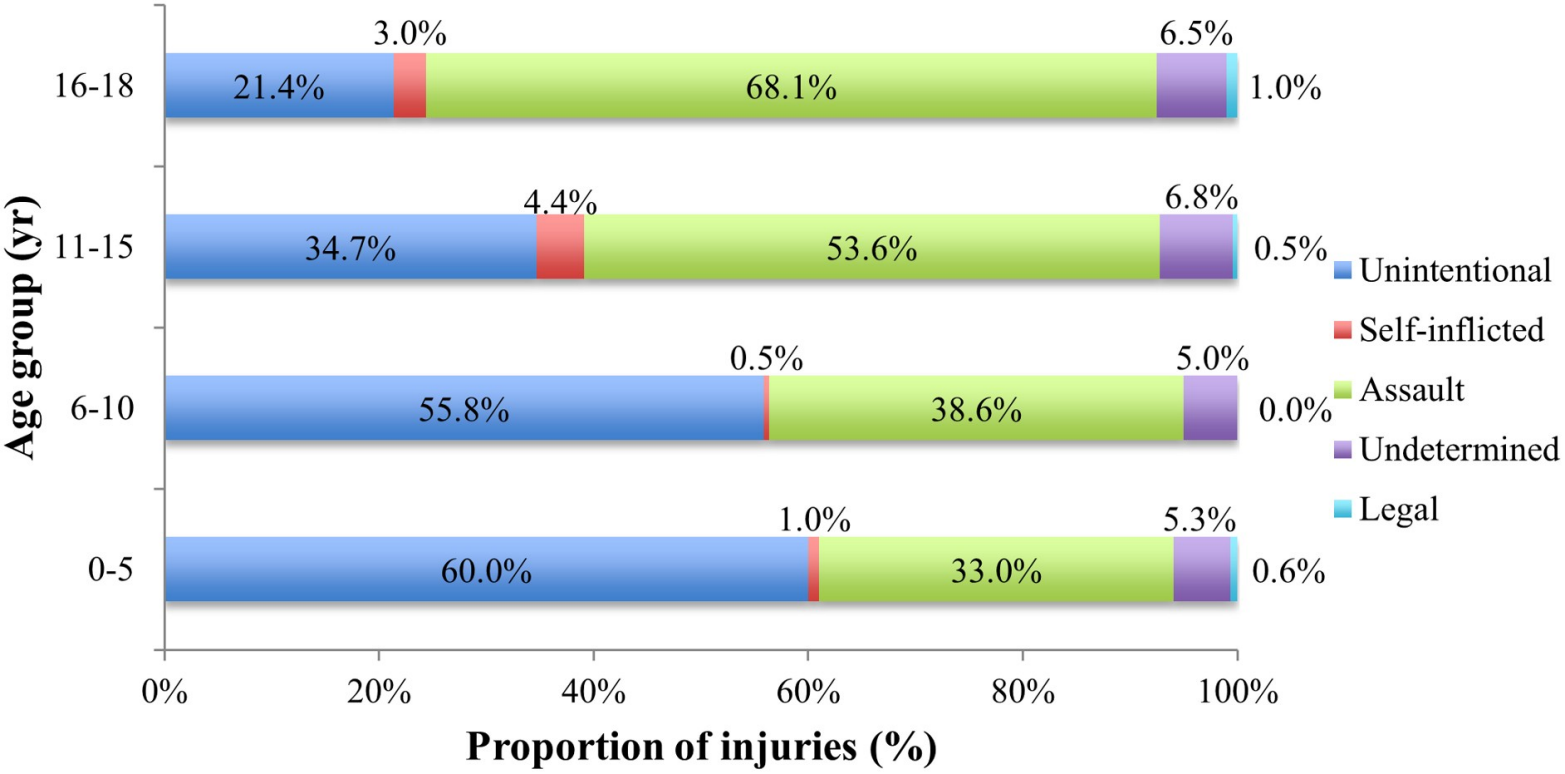
Intent of pediatric firearm-related injuries by age group.

**Fig 3:**
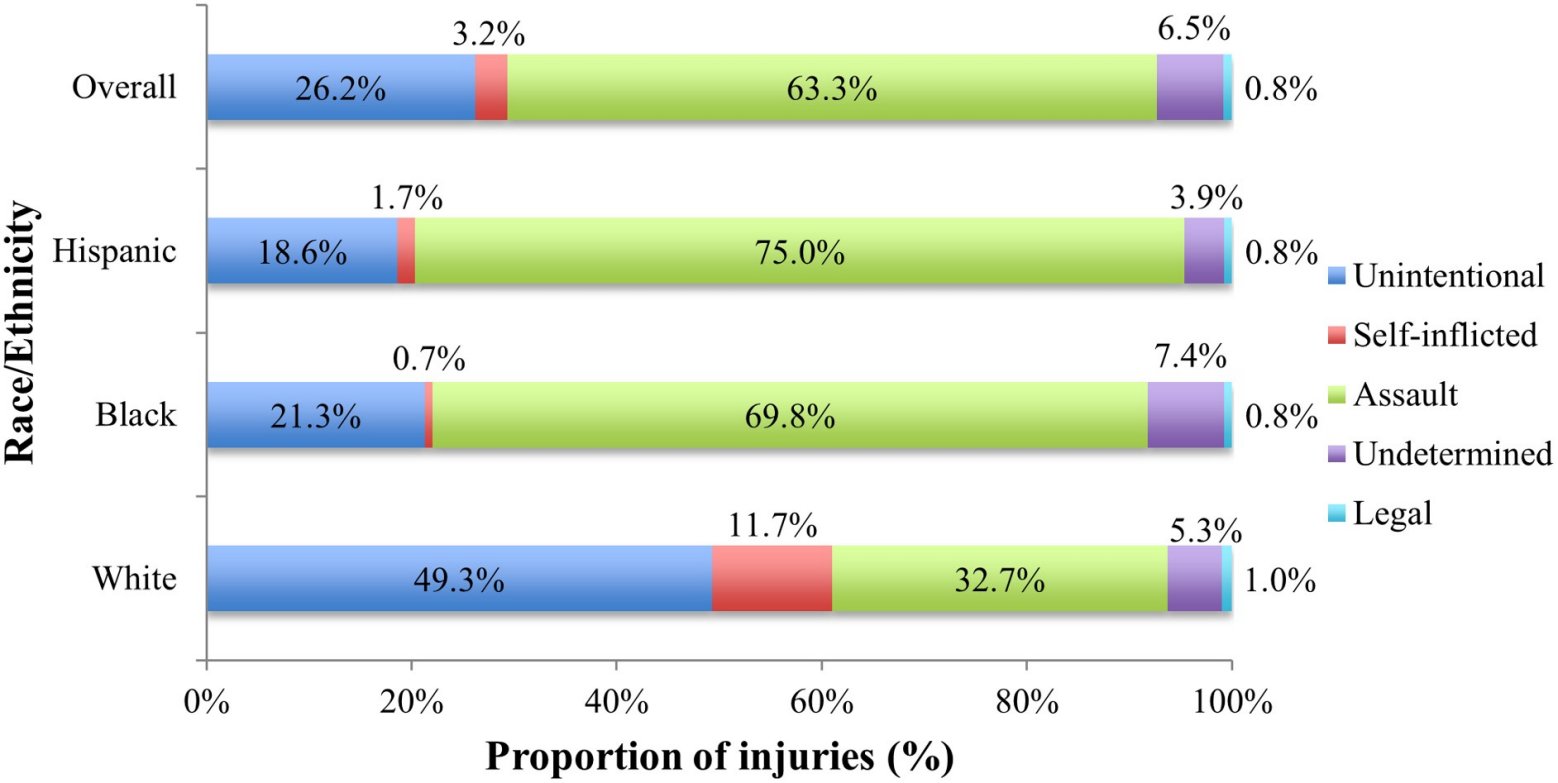
Intent of pediatric firearm-related injuries by race.

When analyzing pediatric firearm-related admissions by race, gender, intent, LOS, disposition, primary payer type, and hospital region, the incidence differs significantly between the age groups (p < 0.001, Table 2). Although the patients were majority male across all age groups, the gender disparity was higher within older age groups. The intent of the injury in younger patients was more likely to be unintentional, whereas assault was more common among older age groups (Fig 2). The youngest age group (0–5 years) had a lower proportion of Medicaid patients than the older age groups. Among the regions, the West had the highest proportion of early childhood injuries (0–5 years). The average white patient was younger and from a higher income quartile than a black or Hispanic patient. The intent of the injury varied significantly between the races, with 49% of injuries to white patients being unintentional, compared to 21% and 19% for blacks and Hispanics, respectively (Fig 3).

**Table 2:** Incidence and median cost of firearm hospitalizations in the U.S. by age (2003, 2006, 2009, 2012).

Dimension	Age groups (years)				P
	Cases, n (%)				
	Median admission cost [IQR], thousands of $				
	0–5	6–10	11–15	16–18	
*Demographic factors*					
**Race**					<0.001
Asian	3 (0.8%)	9 (2.1%)	45 (1.4%)	123 (1.1%)	
	12.7 [12.6, 16.2]	14.6 [12.4, 20.8]	10.8 [5.8, 21.5]	22.9 [11.2, 41.0]	
Black	196 (48.8%)	158 (38.1%)	1563 (49.5%)	6636 (57.5%)	
	12.1 [5.7, 31.9]	12.4 [4.3, 26.1]	12 [5.6, 26.2]	12.8 [6.2, 24.3]	
Hispanic	71 (17.6%)	72 (17.2%)	668 (21.1%)	2632 (22.8%)	
	18.8 [7.1, 39.9]	19.5 [10.7, 34.8]	14.9 [7.3, 32.1]	15.1 [7.5, 33.0]	
Native American	3 (0.8%)	4 (1.0%)	23 (0.7%)	77 (0.7%)	
	1.5 [1.5, 9.1]	7.9 [5.4, 20.4]	8.7 [4.7, 19.8]	12.8 [7.0, 23.0]	
Other	19 (4.7%)	21 (5.1%)	117 (3.7%)	424 (3.7%)	
	10.8 [5.7, 24.5]	14.4 [7.0, 25.8]	15.8 [6.5, 23.0]	14.1 [7.2, 28.1]	
White	110 (27.3%)	151 (36.5%)	744 (23.5%)	1641 (14.2%)	
	14.6 [8.7, 29.5]	13.1 [6.0, 29.9]	13.1 [6.2, 26.7]	14.4 [7.0, 29.7]	
**Gender**					<0.001
Male	315 (63.3%)	363 (70.4%)	3308 (85%)	12626 (91.1%)	
	11.7 [6.2, 27.6]	13.2 [6.2, 26.9]	12.4 [6.0, 25.7]	13.2 [6.6, 25.8]	
Female	183 (36.7%)	153 (29.6%)	583 (15%)	1235 (8.9%)	
	17.9 [6.8, 41.5]	11 [5.3, 23.3]	13.9 [6.3, 27.1]	13.8 [6.5, 26.5]	
**Hospital Region**					<0.001
Midwest	101 (26.1%)	100 (23.8%)	832 (28.6%)	2666 (24.7%)	
	11.2 [6.3, 29.9]	14.1 [5.7, 26.1]	12.3 [6.4, 23.5]	12.8 [6.3, 23.0]	
Northeast	25 (6.5%)	46 (11.0%)	424 (14.6%)	1806 (16.7%)	
	10.2 [3.4, 30.5]	7.5 [2.3, 24.9]	7.7 [3.9, 16.5]	10.3 [4.8, 19.6]	
South	186 (47.7%)	148 (35.4%)	944 (32.4%)	3496 (32.4%)	
	13.3 [5.7, 31.8]	14.8 [6.5, 33.1]	12.7 [6.1, 23.7]	12.8 [6.8, 24.2]	
West	77 (19.7%)	125 (29.9%)	711 (24.4%)	2839 (26.3%)	
	17.8 [9.5, 38.7]	12.1 [6.7, 26.1]	17.8 [8.8, 38.0]	18.5 [9.5, 40.0]	
**Payer**					<0.001
Medicaid	343 (68.8%)	352 (60.3%)	2279 (58.6%)	7337 (52.6%)	
	13.2 [6.2, 32.1]	13 [5.7, 26.9]	12.9 [5.9, 27.2]	13.3 [6.6, 26.4]	
Other	32 (6.3%)	33 (5.7%)	193 (5%)	905 (6.5%)	
	12.7 [5.7, 24.1]	8.2 [2.9, 28.0]	15.7 [7.1, 28.5]	17 [7.7, 34.8]	
Private Insurance	91 (18.2%)	165 (28.3%)	1124 (28.9%)	3910 (28%)	
	15.1 [7.3, 28.7]	11.5 [5.4, 21.5]	12.3 [6.3, 23.6]	13.8 [6.9, 26.3]	
Self-pay	33 (6.7%)	33 (5.7%)	294 (7.6%)	1807 (12.9%)	
	10.2 [5.9, 15.9]	9.6 [6.4, 13.4]	8.5 [4.9, 17.2]	10 [5.1, 18.0]	
**Income quartile**					1
1	258 (53.2%)	276 (47.9%)	2043 (54.0%)	7204 (53.0%)	
	14.1 [6.7, 32.3]	11.6 [5.7, 24.1]	11.4 [5.4, 24.1]	12.3 [6.1, 23.7]	
2	127 (26.2%)	182 (31.7%)	923 (24.4%)	3321 (24.5%)	
	9.3 [5.1, 30.0]	11 [5.1, 23.7]	13.6 [6.7, 27.8]	13.9 [7.3, 26.2]	
3	71 (14.7%)	79 (13.7%)	569 (15.1%)	2103 (15.5%)	
	15.4 [7.9, 27.3]	13.9 [5.4, 22.8]	14.8 [7.7, 27.7]	14.3 [7.1, 29.6]	
4	28 (5.9%)	39 (6.7%)	245 (6.5%)	954 (7.0%)	
	17.3 [10.6, 20.7]	11.5 [5.4, 26.9]	13.2 [6.2, 26.4]	17.3 [7.9, 36.5]	
*Injury Specific factors*					
**ISS**					0.436
0–9	298 (59.5%)	373 (63.8%)	2494 (63.9%)	8612 (61.4%)	
	9.4 [4.9, 20.2]	8.2 [4.0, 15.2]	8.5 [4.4, 16.5]	9.5 [4.8, 17.2]	
10–15	92 (18.5%)	90 (15.4%)	610 (15.6%)	2491 (17.8%)	
	18.7 [9.4, 30.4]	19.2 [10.6, 39.6]	17.9 [10.6, 32.4]	17.3 [10.3, 29.4]	
16–25	85 (17.0%)	93 (16.0%)	621 (15.9%)	2260 (16.1%)	
	31.9 [13.2, 73.6]	23.1 [12.2, 45.1]	24.1 [14.1, 52.9]	24.7 [13.6, 50.2]	
>26	25 (5.0%)	28 (4.8%)	179 (4.6%)	664 (4.7%)	
	29.7 [17.8, 76.7]	30.5 [20.5, 117.3]	40.3 [20.6, 77.4]	37.1 [20.5, 71.8]	
**Intent**					<0.001
Assault	154 (33.0%)	216 (38.6%)	1978 (53.6%)	8996 (68.1%)	
	16.2 [6.2, 36.8]	13.2 [5.9, 24.3]	13.5 [6.3, 28.0]	13.4 [6.7, 25.6]	
Legal Intervention	3 (0.6%)	0 (0.0%)	17 (0.4%)	131 (1.0%)	
	6.00 [6.0, 17.0]	0.0 [0.0, 0.0]	14.3 [10.7, 45.2]	15 [6.9, 48.2]	
Self-inflicted	5 (1.0%)	3 (0.5%)	163 (4.4%)	397 (3.0%)	
	6.1 [4.3, 13.9]	7.9 [7.9, 27.4]	14.2 [7.3, 21.8]	15.7 [7.8, 35.8]	
Undetermined	25 (5.3%)	28 (5.0%)	252 (6.8%)	860 (6.5%)	
	11.4 [6.0, 18.9]	13.9 [4.0, 23.1]	10.8 [5.2, 23.3]	13.1 [6.1, 23.5]	
Unintentional	280 (60.0%)	312 (55.8%)	1279 (34.7%)	2819 (21.3%)	
	11.6 [6.1, 29.9]	10.8 [5.1, 22.4]	10.4 [5.2, 21.7]	11 [5.7, 21.3]	
**LOS, days**					0.030
0	32 (6.4%)	43 (7.3%)	281 (7.2%)	1238 (8.8%)	
	9.6 [2.5, 13.2]	5.5 [2.3, 9.6]	5.1 [2.8, 10.3]	5.2 [2.8, 10.3]	
1	81 (16.1%)	119 (20.4%)	839 (21.5%)	2698 (19.2%)	
	5.2 [3.8, 8.1]	4.3 [2.7, 6.6]	5.6 [3.1, 9.4]	5.5 [3.1, 9.5]	
2	80 (15.9%)	93 (15.9%)	599 (15.3%)	1981 (14.1%)	*
	6.2 [4.1, 9.4]	7.4 [4.4, 10.1]	7.1 [4.2, 11.2]	8.5 [5.4, 12.7]	
3	56 (11.1%)	52 (8.9%)	424 (10.9%)	1433 (10.2%)	
	9.4 [6.8, 16.7]	10 [7.8, 23.2]	10.3 [7, 15.2]	10.8 [7.3, 15.7]	
4+	252 (50.4%)	278 (47.5%)	1761 (45.1%)	6675 (47.6%)	
	29.1 [16.5, 50.8]	23 [14.3, 44.4]	26 [15.6, 49.4]	24.3 [15, 43.5]	
**Disposition**					<0.001
Died in hospital	37 (7.4%)	33 (5.7%)	276 (7.1%)	921 (6.6%)	
	13.2 [9.2, 21.1]	11.9 [8.6, 35.6]	16.7 [9.5, 27.7]	15.8 [9.2, 30.1]	
Home health care	21 (4.2%)	26 (4.5%)	232 (6.0%)	895 (6.4%)	
	19.7 [9.2, 37.8]	20.4 [9.9, 26.2]	25.8 [15.4, 48]	22.2 [12, 42.1]	
Left AMA	2 (0.3%)	0 (0.0%)	11 (0.3%)	139 (1%)	
	8.7 [0.0, 8.7]	0.0 [0.0, 0.0]	7.7 [2.1, 32.8]	8 [4.1, 16.2]	
Routine	398 (79.7%)	460 (78.9%)	3041 (78.1%)	10734 (76.8%)	*
	10.7 [5.9, 26.4]	10 [4.6, 21.3]	10.2 [5, 20.4]	11.4 [5.8, 21.5]	
Transfer to other facility	31 (6.2%)	33 (5.7%)	211 (5.4%)	890 (6.4%)	*
	67.2 [43.8, 101.4]	39 [23.6, 92.7]	48.3 [22.5, 86.6]	38.8 [17.5, 81.4]	
Transfer to short-term hospital	10 (2.1%)	31 (5.2%)	121 (3.1%)	406 (2.9%)	
	22.1 [7.7, 30.8]	13 [9.1, 24.1]	25.7 [13.9, 61.2]	24.2 [9.6, 53.2]	

ISS, injury severity score; LOS, length of stay; AMA, against medical advice; asterisk (*) indicates significant cost variation (p<0.05) based on listed dimension and age group. All other cost variation was not significant.

When analyzing the average costs of admissions by the different age groups, we found that younger patients with LOS of two days or requiring transfer to another hospital had significantly higher hospital costs compared to their older counterparts (Δ$2,414 median cost, p<0.001 and Δ$27,027 median cost, p<0.001, respectively).

## Discussion

Firearm-related injuries among children that are less than or equal to 18 years of age and represent a significant public health problem and financial burden. The cost of initial hospitalization for pediatric firearm-related injuries totaled $436 million over the four years reported, averaging $109 million annually and nearly $13,000 per incidence. The average hospitalization cost (using inflation-adjusted 2018 dollars) per incidence increased between 2003 and 2009 but did not significantly change between 2009 and 2012, a trend that was consistent for patients of all income quartiles and payer types. There was, however, significant cost variation related to patient demographics, most of which was accounted for by a patient’s ISS and hospital LOS. We also found significant cost variation based on hospital region.

The US does not have a single-payer, universal healthcare program and thus the cost of medical care in the US is borne by several financial sources: government-based insurance programs (such as Medicare, Medicaid, and the Veterans Health administration), private insurance (purchased by employers for their employees or by individuals), and out-of-pocket expenses for uninsured individuals [10]. Spitzer et al. found that the government is responsible for 41% of initial hospitalization costs for firearm-related injuries of all ages [4]. We found that the government (Medicaid, which funds low-income and disabled Americans) was responsible for the greatest share of the cost in pediatric patients (57%, >$62 million annually). Medicaid patients also had a high cost per incidence, with a median cost of $13,100. Self-pay patients accounted for 11.5% of admissions, with a median cost of almost $10,000, and 79.6% of all self-pay patients fell below the 50% income percentile. These results suggest that pediatric firearm-related injuries place a disproportionate financial burden on the government and the poor.

Self-inflicted wounds accounted for just over 3% of admissions, incurring a total cost of $31.9 million over the study period. As mentioned, this estimate only includes children who were admitted and does not account for costs associated with emergency room visits. Given that suicide by firearm is highly lethal, with a case fatality rate of 75–96% in children, there are likely many patients who did not survive long enough to be admitted to the hospital and thus were excluded from this analysis [10, 11]. In 2016, firearms were the mechanism of death in 43% of pediatric suicides, and suicide by firearms accounted for 34.9% of all pediatric firearm deaths [3]. For those patients who do survive their self-inflicted wounds to admission, there is a higher median cost per incidence ($15,300) compared to assault victims ($13,400). After controlling for ISS, LOS, and hospital region, this significant difference in cost per incidence disappears. Our analysis likely significantly underestimates the financial burden of self-inflicted pediatric firearm injuries based on the lethality of suicide by firearm and exclusion of patients that do not survive to admission.

While numerous studies have examined the financial burden of firearm-related injuries or fatalities, they often include both adults and children [4, 5, 12–14]. This is the first study that examines recent trends and costs associated with initial pediatric firearm-related hospitalization alone. A better understanding of the scope of the problem may help to focus policy efforts to areas that will have the greatest impact. We found that the initial hospitalization costs for pediatric firearms injures was approximately $109 million per year, with over half of the financial burden being borne by the US government. Reducing the overall cost of firearm-related injuries must involve reducing the incidence of firearm-related injuries as well as the cost of treating individual patients. This study may help to identify interventions that would be the most effective at reducing cost. As an example, since we found that self-inflicted injuries and those resulting from legal intervention were the most costly, policies that aim to decrease the incidence of those acts are may have greater impact in reducing costs. Educational programs and laws stressing safe storage of firearms and child access prevention laws have been demonstrated to reduce pediatric suicide rates and may thus contribute to decreasing the financial burden of pediatric firearms injury as well [15].

The overall cost of firearm-related hospitalization is inflated by significant cost variation based on demographics and hospital region. Patients in the West had a median cost of at least $5,405 more per hospital admission than any other region. This may be accounted for, in part, for a higher average ISS in the West compared with other regions. Other factors, however, are likely to contribute to this cost discrepancy, including the cost of living. In 2006–2010, 7 of the 10 metropolitan areas with the highest cost of living (as measured by the regional price parity) were in the West [16]. Though reasons for cost variation are multifactorial, the predominant contributor to cost variation is arguably variability in how hospitals and providers approach patient care with respect to available technologies and resources [17]. As an example, we found that younger patients (0–5 years) incurred significantly higher costs per admissions, even after selecting for the lowest ISS and shortest LOS. The higher cost may be due, in part, to the increased variation in patient care (i.e. number and frequency of diagnostic tests) from providers who are not accustomed to managing firearm-related injuries in this age group; young children (0–5 years) account for only 2.6% of pediatric firearm-related injuries. Such variation could potentially be improved by standardizing diagnostic and management practices when appropriate or early referral and transfer of care to pediatric trauma centers. Further investigation is needed to examine the driving factors behind the significantly higher hospitalization costs in the West and among younger patients for pediatric firearm-related injuries which are not beyond the granularity of the KID database.

These study findings represent a comprehensive national sampling, however, there are limitations to how the data should be extrapolated. The costs reported represent initial hospitalization, and do not account for costs from subsequent admissions, costs from those patients who are discharged directly from the emergency department, or those who succumb to their injuries prior to admission. Furthermore, the costs do not include additional financial burdens like lost time from work for both teens and parents, rehabilitation, long-term disability costs, lost productivity from premature death, and non-hospital resources like the cost of first responders and police involvement.

Future studies can address the limitations to the present study to better illustrate a more holistic estimation of the cost of pediatric firearm violence on society. Moreover, additional costs that must be considered include the loss of productivity for a parent when a child is injured or lost, as well as the potential of contribution of that is lost from the injured or killed child.

## Conclusions

Firearm-related hospitalizations in pediatric patients are a significant financial burden on healthcare systems. The average cost of initial hospitalization is even when controlled for inflation. The overall financial and societal costs of pediatric firearm injuries extends well beyond initial hospitalization costs. Failure to contain such costs through meaningful prevention programs and firearms legislation will continue to burden an already overloaded US healthcare system.
